# ALIGNing-CARE in Geriatric Surgery Co-Management: Pre-operative Phase

**DOI:** 10.1093/geroni/igaf122.2002

**Published:** 2025-12-31

**Authors:** Stephanie Chow, Yihan Wang, William Hung, Abigail Baim-Lance, Ko Harada, Danialle Coyne, Fred Ko

**Affiliations:** Icahn School of Medicine at Mount Sinai, New York, New York, United States; Mount Sinai Hospital, New York, New York, United States; James J Peters VA Medical Center, New York, New York, United States; Veterans Health Administration, New York, New York, United States; Icahn School of Medicine at Mount Sinai, New York, New York, United States; Mount Sinai Hospital, New York, New York, United States; James J. Peters Department of Veterans Affairs Medical Center, New York, New York, United States

## Abstract

Older patients comprise more than half of surgeries, with greater risk for death, loss of function and quality of life. Geriatric Surgery Co-Management programs aim to assess peri-operative risks and improve surgical outcomes, via comprehensive assessments and interprofessional interventions. Our ALIGN-CARE pilot explored the outcomes and potential for pre-operative intervention in older frail adults. Surgical candidates 65+ years screened pre-frail or frail by surgeons were included. Enrolled patients underwent a comprehensive geriatric and frailty assessment, and an ALIGN-CARE framework of measurements (“7Ms”) to assess and intervene on important geriatric domains. Patients were offered pre-operative interventions and followed to surgery. Data was collected from January 2022 to May 2024. ALIGN-CARE enrolled 51 patients, mean age 78.5 years, 45.1% female, 49% reported primary spoken language other than English. Surgeons from 7 specialties were included with Urology comprising majority (58.5%) of referrals. Of those enrolled, 42% were clinically frail. Of the 7Ms framework, recommendations and interventions included 19% of PCPs outreached by ALIGN-CARE, 50.8% recommended for medication adjustment, 54% advised for physical therapy or pre-rehabilitation, 15.9% newly diagnosed with MCI or dementia, 22.2% recommended for further cognitive testing, 46.0% completed healthcare proxy form, 20.6% discussed advance care planning, and 44.4% scheduled for a social work visit. ALIGN-CARE assessment and interventions spanned important domains of geriatric care, signaling need for interprofessional incorporation of medical and psychosocial care in older frail persons. Further data on patient and provider experience in this co-management model, combined with extension into post-operative phases of co-management, is needed.

